# The Effect of Chronic Hepatitis B Virus Infection on BDCA3+ Dendritic Cell Frequency and Function

**DOI:** 10.1371/journal.pone.0161235

**Published:** 2016-08-16

**Authors:** Evelyn van der Aa, Sonja I. Buschow, Paula J. Biesta, Harry L. A. Janssen, Andrea M. Woltman

**Affiliations:** Department of Gastroenterology and Hepatology, Erasmus MC-University Medical Center, Rotterdam, The Netherlands; The University of Melbourne, AUSTRALIA

## Abstract

Chronic hepatitis B virus (HBV) infection results from inadequate HBV-specific immunity. BDCA3^+^ dendritic cells (DCs) are professional antigen presenting cells considered to be important for antiviral responses because of specific characteristics, including high interferon-λ production. BDCA3^+^ DCs may thus also have a role in the immune response against HBV, and immunotherapeutic strategies aiming to activate DCs, including BDCA3^+^ DCs, in patient livers may represent an interesting treatment option for chronic HBV. However, neither the effect of chronic hepatitis B (CHB) infection on the frequency and function of BDCA3^+^ DCs in liver and blood, nor the effect of the viral surface protein (HBsAg) that is abundantly present in blood of infected individuals are known. Here, we provide an overview of BDCA3^+^ DC frequency and functional capacity in CHB patients. We find that intrahepatic BDCA3^+^ DC numbers are increased in CHB patients. BDCA3^+^ DCs from patient blood are not more mature at steady state, but display an impaired capacity to mature and to produce interferon-λ upon polyI:C stimulation. Furthermore, in vitro experiments exposing blood and intrahepatic BDCA3^+^ DCs to the viral envelope protein HBsAg demonstrate that HBsAg does not directly induce phenotypical maturation of BDCA3^+^ DCs, but may reduce IFN-λ production via an indirect unknown mechanism. These results suggest that BDCA3^+^ DCs are available in the blood and on site in HBV infected livers, but measures may need to be taken to revive their function for DC-targeted therapy.

## Introduction

Hepatitis B virus (HBV) specifically infects hepatocytes and can cause chronic liver infection, often leading to severe liver diseases [1]. Chronic viral infection results from inadequate antiviral immunity, however, the mechanisms underlying ineffective HBV-specific immunity remain poorly understood [2, 3]. Effective viral immunity includes induction of antiviral cytokines such as interferons (IFNs) and virus-specific CD8^+^ cytotoxic T lymphocytes (CTL). Dendritic cells (DCs) play a crucial role in this process because they can, uniquely, activate virus-specific naïve T cells and produce high type I and type III IFN levels [4, 5]. The DC family comprises several subsets, including plasmacytoid DCs (pDC) and the myeloid DC (mDC) subsets BDCA1^+^ DCs and BDCA3^hi^CLEC9A^+^ DCs [6–8]. These DC subsets differ in ontogeny, localization, phenotype and function.

We and others have previously characterized the frequency and function of BDCA1^+^ DCs and pDCs in peripheral blood of chronic HBV patients [9]. We demonstrated that although DC frequencies in blood were unaffected, blood BDCA1^+^ DCs were impaired in their capacity to mature, to produce pro-inflammatory cytokines and to stimulate T cells, and that pDCs were impaired in IFNα-producing capacity [10, 11]. More recently, BDCA3^hi^CLEC9A^+^ DCs (further referred to as BDCA3^+^ DCs) were identified and shown to excel over other DC subsets in cross-presentation of cell-associated antigens (Ag) to CD8^+^ T cells [12–15]. In mice, the equivalents of BDCA3^+^ DCs (CD8α^+^ and CD103^+^ DCs) have been shown to be crucial for generating optimal virus-specific CD8^+^ T cell responses to influenza virus and West Nile virus [16, 17]. In addition, BDCA3^+^ DCs are the most potent producers of IFN-λ in response to viruses that induce TLR3 signaling, or in response to the synthetic RNA polyI:C [18–22]. IFN-λ is an important antiviral cytokine that supports T cell skewing towards Th1 responses and has antiviral activity against multiple viruses, including HBV [23, 24]. Although the effect of IFN-λ on HBV replication in *in vitro* and mouse studies was debatable [25, 26], a recent clinical trial showed that in HBeAg-positive patients, PEG-IFN-λ induced a clear reduction in HBV DNA and viral surface antigen (HBsAg) levels, indicating that this cytokine may be valuable to fight CHB, and we envision that this cytokine could be even more effective when secreted on site [27]. BDCA3^+^ DCs may thus be a viable target to induce an effective immune response against HBV. BDCA3^+^ DCs are known to be present in human liver [21, 28, 29], however, it is unknown whether this is altered upon HBV-infection. Furthermore, the actual localization of BDCA3^+^ DCs within healthy and diseased liver tissue, as well as their functional state in HBV patients, and their response to the abundantly circulating HBsAg remains elusive.

We here assessed the presence of BDCA3^+^ DCs in liver and blood of HBV-infected patients, as well as the effect of chronic HBV infection and HBsAg on BDCA3^+^ DC phenotype and function in vitro and ex vivo. We found that although BDCA3^+^ DCs are present in the liver immune infiltrate of chronic HBV (CHB) patients, their function may be compromised.

## Materials and Methods

### Patients and controls

Heparinized peripheral blood samples were obtained from CHB patients and control subjects of which the clinical characteristics are listed in Table 1. CHB patients and control subjects used for functional characterization of DCs were matched for age, gender and race. Liver tissue was obtained from HBV-infected livers and non-HBV-infected livers, i.e. donor livers that were used for transplantation, donor livers that were rejected for transplantation, or non-cancerous peri-tumor tissue of donors who had not received chemotherapy in at least three months prior to tissue donation. The clinical characteristics of donors are summarized in Table 2. All patients were negative for antibodies (Abs) against hepatitis C, hepatitis D and human immunodeficiency virus. Patients did not receive antiviral therapy at time of blood or tissue donation. The study was approved by the medical ethical committee of the Erasmus MC University Medical Center and donors gave written informed consent before blood or tissue donation.

**Table 1:** Characteristics of donors used for peripheral blood DC quantification and functional characterization.

Characteristics	Quantification	Functional characterization
Number	19	18
ALT (IU/L) mean (range)	49 (12–164)	37 (12–78)
HBV DNA (log10 IU/ml) mean (range)	3.9 (1.3–8.6)	3.5 (1.3–8.6)
HBeAg+/HBeAg-	2/17	0/18

**Table 2:** Characteristics of donors used for intrahepatic DC quantification.

Characteristics	Flow cytometry	Immunohistochemistry
Number	11	14
ALT (IU/L) mean (range)	73 (32–164)	91 (12–370)
HBV DNA (log10 IU/ml) mean (range)	3.8 (1.3–8.6)	4.6 (1.3–9.5)
HBeAg+/HBeAg-	3/8	10/4
Fibrosis		
F0	1	3^a^
F1	6	6
F2	2	2
F3	2	2

^a^ Fibrosis status of one patient is unkown.

### Cell isolation and stimulation

Peripheral blood mononuclear cells (PBMC) were isolated from heparinized peripheral blood samples or buffy coats from healthy blood donors using Ficoll density gradient centrifugation. PBMC were enriched for DCs using Dynabeads that deplete T cells, B cells, monocytes/macrophages, NK cells, erythrocytes and most granulocytes (Life Technologies), and DC subsets were sorted based on BDCA3 expression using a FACSAria (BD Biosciences).

Liver tissue (>1 cm^3^) was digested to obtain a single cell suspension. Briefly, liver tissue was cut into small pieces and digested with 0.5 mg mL^-1^ collagenase (Sigma-Aldrich) and 0.1 mg ml^-1^ DNase (Roche) for 30 minutes at 37°C. The digested material was subsequently filtered through a cell strainer and mononuclear cells were obtained by Ficoll density gradient centrifugation. Core needle-biopsies (14-gauge needle) were only filtered through a cell strainer to obtain a single cell suspension.

### Flow cytometric analysis

For phenotypic analysis, cells were labelled with Abs recognizing CD11c (3.9), CD40 (5C3), CD45 (HI30), CD83 (HB15e), HLA-DR (LN3, all eBioscience), BDCA1/CD1c (AD5-8E7, Miltenyi Biotec), BDCA3/CD141 (AD5-14H12, Miltenyi Biotec), CD86 (2331, BD Horizon), CLEC9A (8F9, BioLegend), HBsAg (Acris), a lineage cocktail including CD3 (UCHT1, eBioscience), CD14 (61D3, eBioscience), CD19 (HIB19, eBioscience) and CD56 (MY31, BD Biosciences), and the live/dead marker Aqua (LifeTechnologies). Fluorescence was measured using a FACS Canto II (BD Biosciences).

### Cytokine production

1*10^6^ PBMC were stimulated with polyI:C (20 μg ml^-1^, Invivogen) in 250 μl in 96-wells plates (Greiner Bio-one, Alphen aan den Rijn, Netherlands) for 5 or 7 hours at 37°C in RPMI 1640 (Invitrogen) supplemented with 9% heat-inactivated FCS (Sigma-Aldrich) and penicillin/streptomycin (Invitrogen). During the last 3 hours, cells were incubated with 10 μg ml^-1^ Brefeldin A (Sigma-Aldrich). Subsequently, cells were stained for BDCA3 and CD11c, fixed with 2% formaldehyde, permeabilized with 0.5% saponin and stained for tumor necrosis factor α (TNF-α) (eBioscience), IFN-λ1 (kindly provided by Bristol-Myers Squibb and commercial Ab from R&D systems) or polyclonal goat IgG (R&D systems). Cytokine-producing cell frequencies were determined by flow cytometry.

Isolated DCs were stimulated for 24 hours with 20 μg ml^-1^ polyI:C in the presence of 10 ng ml^-1^ GM-CSF. Levels of secreted human IFN-λ1 (interleukin 29; IL-29) were measured using a commercially available ELISA kit (eBioscience) and IL-1β, IL-6, IL-8 and TNFα levels were measured using a BD cytometric bead array (CBA, BD Biosciences). Detection limits were 8 pg ml^-1^ (IFN-λ1), 7.2 pg ml^-1^ (IL-1β), 2.5 pg ml^-1^ (IL-6), 3.6 pg ml^-1^ (IL-8), 3.7 pg ml^-1^ (TNF-α).

### HBsAg uptake/binding and stimulation with HBsAg in vitro

For analysis of HBsAg binding/uptake, PBMC were incubated with 1 μg ml^-1^ fluorochrome-labeled HBsAg isolated from pooled serum of patients (pHBsAg; subtype ay; American Research Products, ARP) in 250 μl for 2 hours at 4°C or 37°C. Cells were fixed with 2% formaldehyde and analysed by flow cytometry. For analysis of the effect of HBsAg on BDCA3^+^ DC function, cells were stimulated with 20 μg ml^-1^ polyI:C in the presence or absence of 5 μg ml^-1^ patient-derived pHBsAg or 5 μg ml^-1^ recombinant CHO-derived HBsAg (rHBsAg; Prospec; determined to be endotoxin free by Endolisa; Hyglos GmbH). Depletion of rHBsAg was performed by immunoprecipitation using protein G sepharose beads (GE Healthcare) that were bound to human anti-HBsAg Abs (Biotest Pharma), as described previously [30].

### Immunohistochemistry

Formalin-fixed paraffin-embedded (FFPE) sections (5 μm) of 14 CHB livers and 6 donor livers (Table 2) were deparaffinized and boiled for 10 minutes in citrate buffer (pH 6) for antigen retrieval. Sections were incubated with 3% H_2_O_2_, 10% human serum, and Abs against CLEC9A (polyclonal sheep, R&D systems) or non-specific polyclonal sheep Abs (R&D systems) for 1 hour at 37°C. CLEC9A Ab was subsequently bound by horseradish peroxidase (HRP)-labeled donkey(Fab)-anti-sheep Ab (Life Technologies) and the signal was amplified using tyramide-FITC, followed by mouse-anti-FITC (Jackson ImmunoResearch) and DyLight488-labeled goat-anti-mouse (BioLegend). CLEC9A^+^ cells were manually counted in 5–9 microscopic fields (200x magnification) containing portal tracts and the mean number of cells per microscopic field was calculated.

### Statistical analysis

Statistical analysis was performed using Graphpad Prism version 5.01 for Windows (GraphPad Software).

## Results

### BDCA3^+^ DCs are prominently present in HBV-infected livers

**Fig 1:**
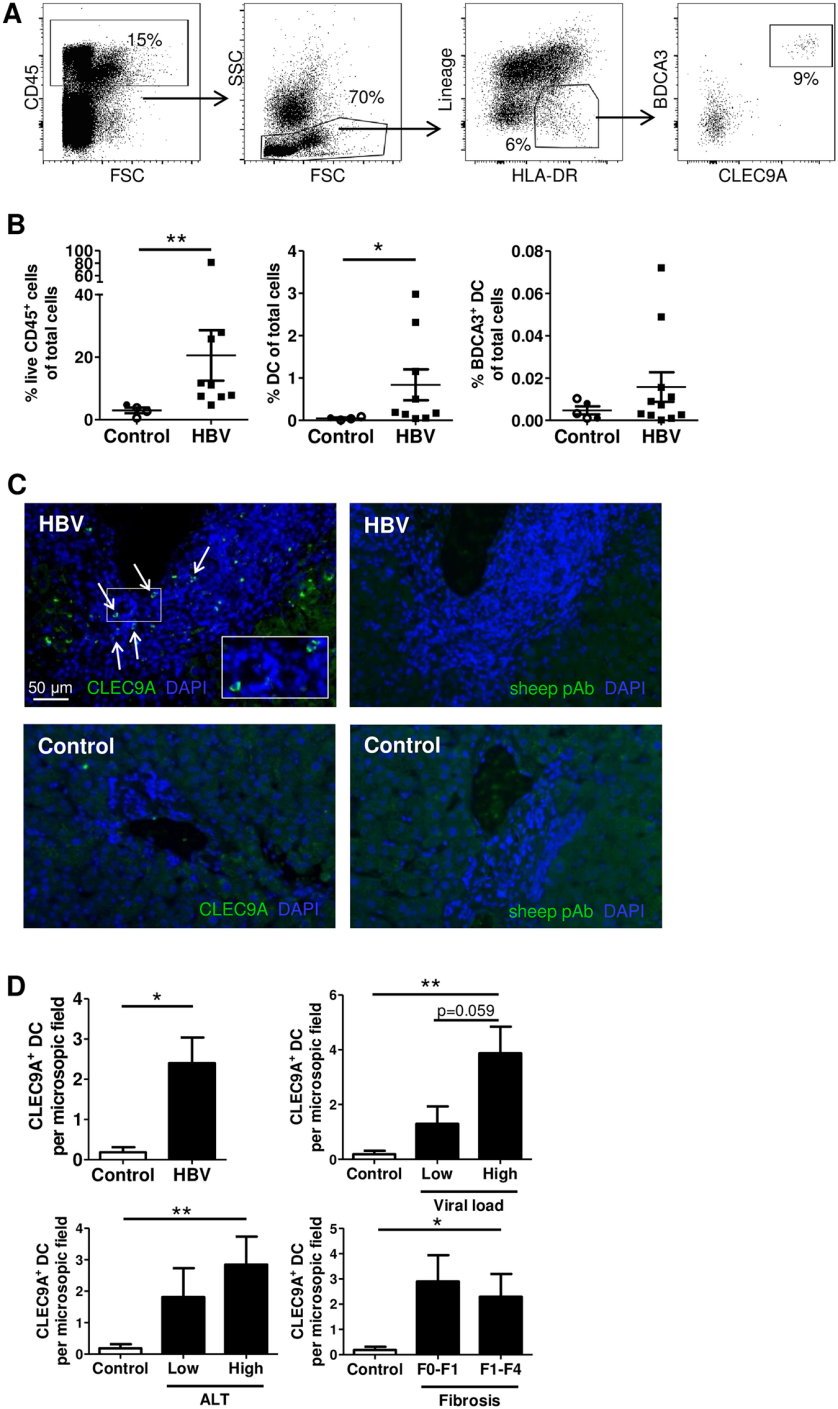
Quantification of intrahepatic BDCA3^+^ DCs from HBV patients and controls. (A-B) Liver cells were isolated from HBV patients and controls. The DC population was identified as CD45^+^Lineage^-^HLA-DR^+^ mononuclear cells, within which BDCA3^+^CLEC9A^+^ DCs were detected. (A) Representative flow cytometry plots and (B) the percentage CD45^+^ cells of total cells (control n = 4, HBV n = 9), percentage DCs of total cells (control n = 4, HBV n = 9) and percentage BDCA3^+^ DCs of total cells (control n = 5, HBV n = 11) in livers of controls and HBV patients. Indicated are the mean percentage and SEM. Open dots represent cells from donor livers and filled dots represent cells from peri-tumor liver tissue. **p < 0.01 by Mann-Whitney test. (C-D) FFPE sections of HBV-infected and control livers were stained with anti-CLEC9A Abs or non-specific sheep polyclonal Abs (green) and quantified (see methods). Nuclei were visualized using DAPI (blue). Magnification 200x. (C) Representative pictures of an HBV-infected liver with high ALT (defined as > 60 IU L^-1^) and high viral load (>10,000 IU ml^-1^) and a control liver (healthy donor liver accepted for transplantation). White arrows indicate CLEC9A^+^ DCs. (D) Number of CLEC9A^+^ DCs per microscopic field in control livers (n = 6) and total HBV-infected livers with different levels of viral load (low n = 8, high n = 6), ALT (low n = 8, high n = 6), and fibrosis (F0–F0-1 n = 6, F1–F4 n = 7) (mean±SEM). *p < 0.05, **p <0.01 by Mann-Whitney test.

The frequency and function of BDCA3^+^ DCs in HBV-infected livers is currently unknown. Therefore, we set out to quantify BDCA3^+^ DCs in biopsies from HBV-infected livers and control livers from non-HBV infected individuals. The latter included healthy donor livers accepted or rejected for transplantation, as well as non-cancerous peri-tumor tissue. First, to distinguish immune cells from hepatocytes, we used the hematopoietic lineage marker CD45 (Fig 1A). As may be expected in a state of ongoing inflammation, the frequency of total CD45^+^ immune cells and DCs was significantly higher in livers of HBV-infected individuals compared to those of controls, indicating extensive immune cell infiltration (Fig 1B). Of these CD45^+^ immune cells, BDCA3^+^ DCs represented 0.18±0.15% both in control livers and HBV-infected livers, which is in line with previous reports (S1 Fig) [29]. In line with an increase of immune cells in the liver, the percentage of BDCA3^+^ DCs of total liver cells seemed to be higher in HBV-infected livers, however, this increase was not significant.

In addition to flow cytometry, immunohistochemical (IHC) stainings were used to accurately study BDCA3^+^ DC frequencies in the liver. This analysis demonstrated that numbers of CLEC9A^+^ DCs were indeed higher in HBV-infected livers than in control livers (Fig 1C and 1D). The marker CLEC9A, that was used to identify BDCA3^+^CLEC9A^+^ DCs by IHC, was uniquely expressed by CD45^+^HLA-DR^+^Lineage^-^BDCA3^hi^ DCs in the liver (S2 Fig). BDCA3^+^ DCs were predominantly located in portal tracts with immune infiltration. Interestingly, BDCA3^+^ DC numbers in the liver positively correlated with HBV DNA levels (Spearman *r* = 0.782, p = 0.001). However, no difference in BDCA3^+^ DC numbers could be detected between patients with high or low fibrosis or liver damage, as measured by alanine transaminase (ALT) (Fig 1D), suggesting that mostly active viral replication, and possibly consecutive local production of inflammatory cytokines/chemokines, rather than liver damage induces infiltration of BDCA3^+^ DCs into the liver. Unfortunately, any association between BDCA3^+^ DC numbers with HBsAg levels could not be determined as HBsAg levels at the timepoint of biopsy collection were not available for all donors.

Together, these data show that during CHB infection, intrahepatic BDCA3^+^ DCs are present at a similar frequency with respect to other immune cells as in control livers. The absolute number of BDCA3^+^ DCs, however, is increased in HBV-infected livers due to the increased inflammatory infiltrate.

### The capacity of blood BDCA3^+^ DCs to mature and produce IFN-λ is reduced in chronic HBV patients

**Fig 2:**
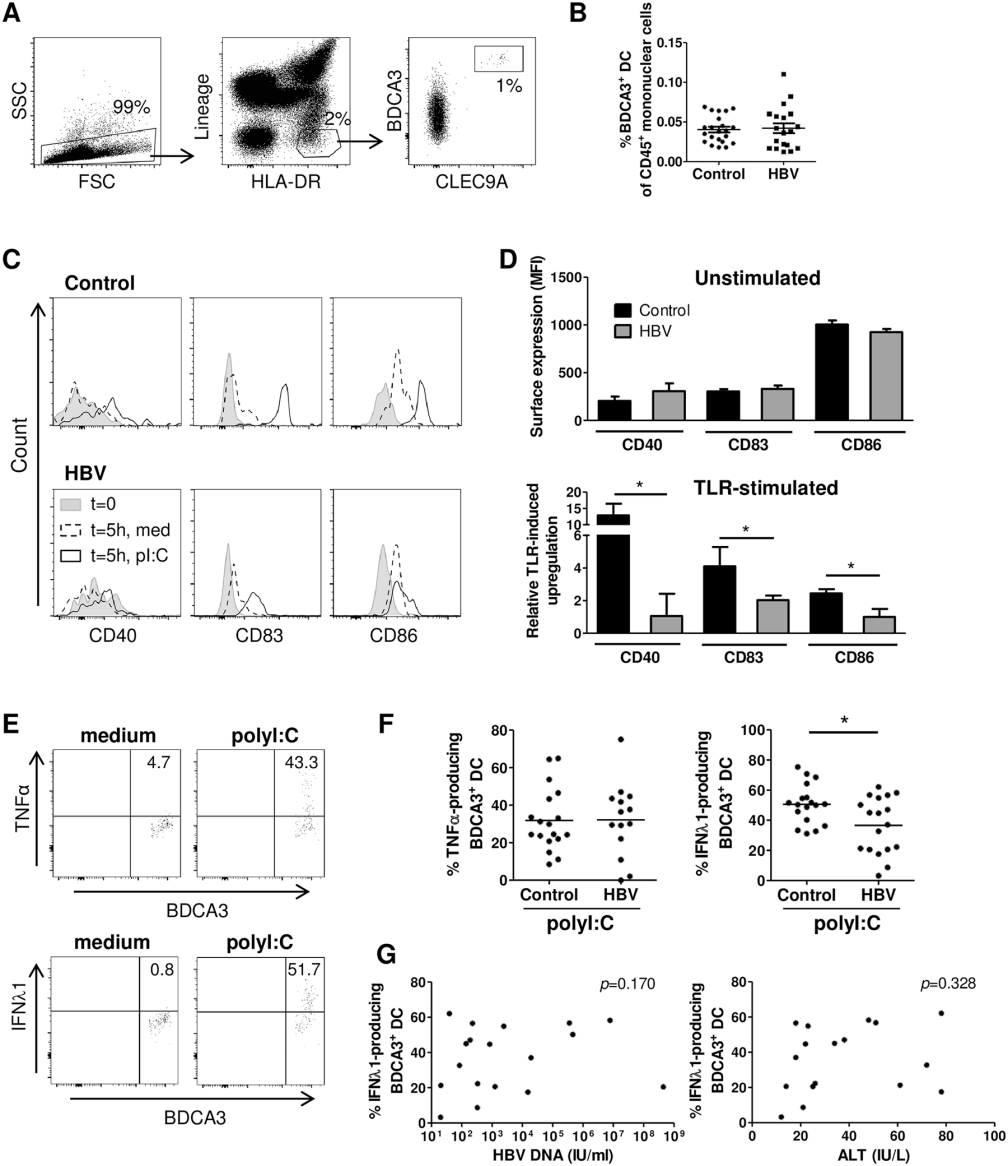
Blood BDCA3^+^ DCs from CHB patients are impaired in their capacity to mature and produce IFN-λ. PBMC were isolated from CHB patients and healthy controls. (A-B) The DC population was identified as Lineage^-^HLA-DR^+^ mononuclear cells, within which BDCA3^+^CLEC9A^+^ DCs were detected. (A) Representative flow cytometry plots and (B) the percentage of BDCA3^+^ DCs of CD45^+^ mononuclear cells (control n = 22, HBV n = 19; mean±SEM). PBMCs were stimulated for 5 hours with or without polyI:C. Expression of the maturation markers CD40, CD83 and CD86, and cytokine production by BDCA3^+^ DCs was measured by flow cytometry. (C) Representative histograms of maturation marker expression by BDCA3^+^ DCs. (D) Collected expression data (MFI) for each marker after isolation at t = 0 (Unstimulated), and relative upregulation after stimulation compared to the medium control at t = 5 (TLR-stimulated) (control n = 15, HBV n = 8) (mean±SEM). (E) Representative flow cytometry plots of TNFα and IFN-λ1 production by FSC/SSC gated viable BDCA3^+^ DC. (F) Collected percentages of TNFα-producing and IFN-λ1-producing BDCA3^+^ DCs in controls (n = 18) and HBV patients (n = 18) (mean±SEM). *p < 0.05 by Mann-Whitney test. (G) Spearman’s correlation between the frequency of IFN-λ1-producing BDCA3^+^ DCs from HBV patients and serum HBV DNA or serum ALT levels (n = 18).

Investigation of the functional state of intrahepatic BDCA3^+^ DCs in CHB patients was not feasible due to the limited amount of biopsy material. Therefore, the possible effects that HBV infection may have on the function of BDCA3^+^ DCs were assessed on BDCA3^+^ DCs from peripheral blood of CHB patients and healthy controls. BDCA3^+^ DCs were equally present in blood of HBV patients and healthy controls (both 0.04%, Fig 2A and 2B). Blood BDCA3^+^ DCs of both HBV patients and controls were largely immature, as indicated by low expression of the maturation markers CD40, CD83 and CD86 (Fig 2C and 2D). Subsequent in vitro maturation by polyI:C induced upregulation of maturation markers both in healthy control DCs and DCs from HBV patients, however, this was much less pronounced in BDCA3^+^ DCs from HBV patients (Fig 2D and S3 Fig). Assessment of cytokine secretion showed that blood-derived BDCA3^+^ DCs produced TNF-α and IFN-λ1, but no IFN-α or IFN-β, upon polyI:C stimulation (Fig 2E and 2F, S4A Fig, data not shown). Most IFN-λ-producing BDCA3^+^ DCs co-produced TNF-α, and IFN-λ production correlated with TNF-α production (S4B and S4C Fig). However, only the secretion of IFN-λ1 by blood BDCA3^+^ DCs from HBV patients was significantly impaired (Fig 2E and 2F). The frequency of IFN-λ1-producing BDCA3^+^ DCs did neither correlate with serum HBV DNA or serum ALT levels nor age (Fig 2G, data not shown). Together, these results indicate that, although BDCA3^+^ DCs are not matured by chronic HBV infection, the capacity of CHB patient-derived blood BDCA3^+^ DCs to mature and produce IFN-λ1 upon TLR activation is impaired.

### HBsAg can affect BDCA3^+^ DC function via an indirect effect

**Fig 3:**
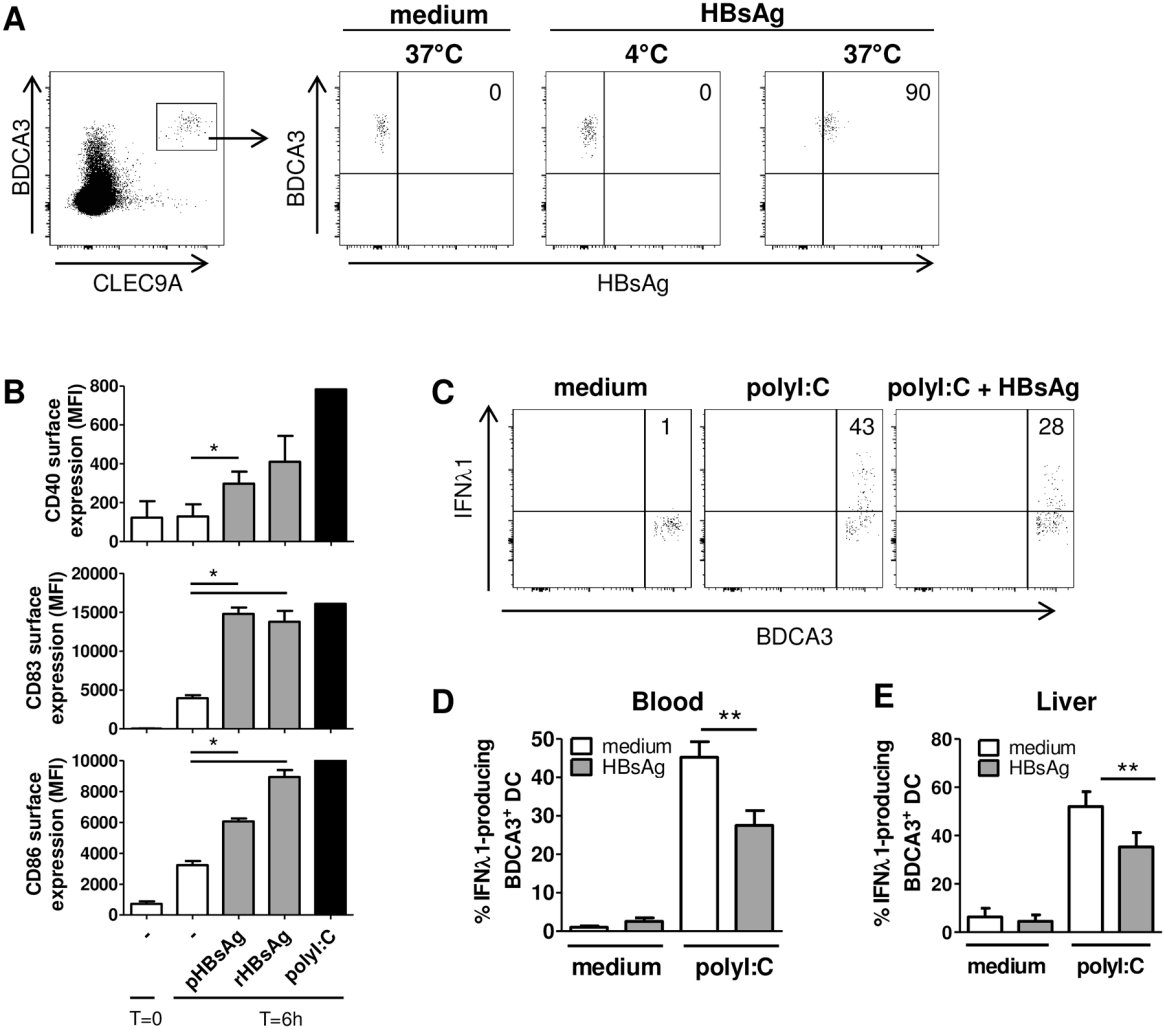
HBsAg diminishes IFN-λ1 production by BDCA3^+^ DCs. (A) PBMC from healthy subjects were incubated with or without fluorescently-labeled HBsAg for 2 hours at indicated temparatures and HBsAg binding/uptake by BDCA3^+^ DCs was measured by flow cytometry. Representative plots of 3 independent experiments and donors are shown. (B) PBMC from healthy subjects were stimulated for 6 hours with or without rHBsAg, pHBsAg or polyI:C and maturation marker-expression on BDCA3^+^ DCs was analyzed by flow cytometry (n = 3; mean±SEM; * p<0.05 by paired Student’s *t*-test). (C-D) PBMC from healthy subjects were stimulated for 7 hours with polyI:C in the presence or absence of rHBsAg and the production of IFN-λ1 by BDCA3^+^ DCs was measured by ICS. Representative flow cytometry plots (C) and the summarized percentage of IFN-λ1-producing BDCA3^+^ DCs (D; n = 7; mean±SEM) are shown. To determine the percentage of IFN-λ-producing BDCA3^+^ DCs in blood, a minimum threshold of 70 BDCA3^+^ DCs was used. **p < 0.01 by paired Student’s *t*-test. (E) Liver cells from peri-tumor liver tissue were stimulated for 5 hours with or without polyI:C in the presence or absence of rHBsAg and the production of IFN-λ1 by BDCA3^+^ DCs was measured by ICS. The percentage of IFN-λ1-producing BDCA3^+^ DCs is shown (n = 3; mean±SEM). **p < 0.01 by paired Student’s *t*-test.

HBsAg, an HBV-derived protein which is abundantly present in patient’s circulation, has previously been shown to functionally impair pDC function [11, 31]. We therefore investigated whether HBsAg affected BDCA3^+^ DC function by incubating BDCA3^+^ DCs with HBsAg in vitro. Using fluorochrome-labeled patient-derived HBsAg (pHBsAg) we observed that BDCA3^+^ DCs readily internalized HBsAg via active endocytosis (Fig 3A). As we used a concentration comparable to that found in vivo, these results indicate that a direct interaction between BDCA3^+^ DCs and HBsAg is also likely to occur in vivo.

**Fig 4:**
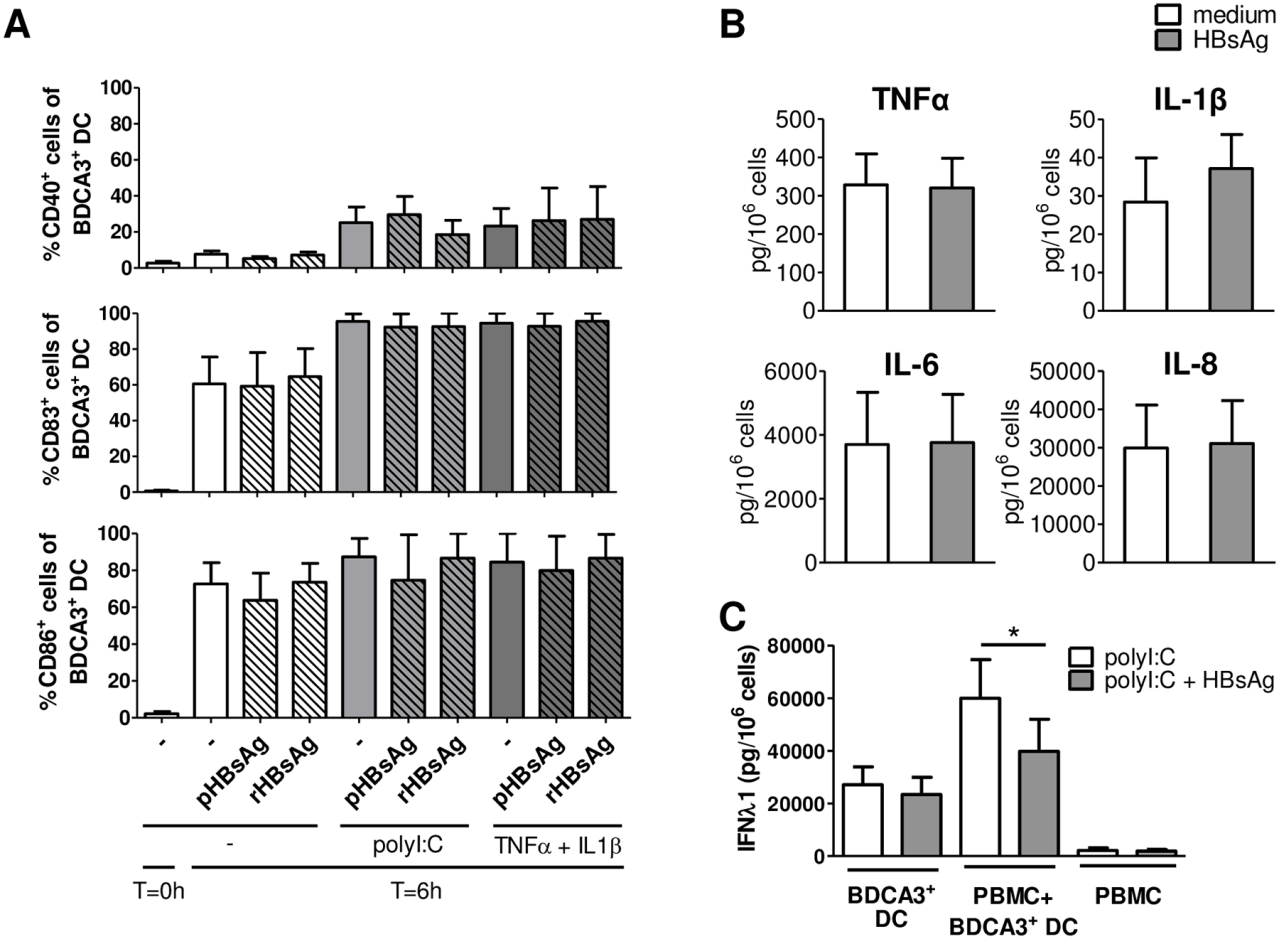
HBsAg does not have a direct effect on BDCA3^+^ DC function. (A) Isolated BDCA3^+^ DCs from healthy subjects were stimulated for 6 hours with or without polyI:C or TNFα and IL-1β in the presence or absence of rHBsAg or pHBsAg. Mean±SEM percentages of maturation marker-expressing BDCA3^+^ DCs are shown (n = 2–3). (B) Isolated BDCA3^+^ DCs from healthy subjects were stimulated for 24 hours with polyI:C in the presence or absence of rHBsAg. Data are shown as mean±SEM cytokine levels determined by CBA (n = 5). (C) Isolated blood BDCA3^+^ DCs from healthy subjects were stimulated for 24 hours with polyI:C in the presence (n = 6) or absence (n = 7) of PMBC and/or rHBsAg, and cytokine levels in the culture supernatant were determined by ELISA (mean±SEM). *p < 0.05 by paired Student’s *t*-test.

Incubation of PBMCs with either pHBsAg or recombinant HBsAg (rHBsAg) for 6 hours increased CD40, CD83 and CD86 expression on BDCA3^+^ DCs, showing that under these circumstances the viral antigen can induce maturation (Fig 3B). Contamination of endotoxins in the HBsAg preparations, which might affect BDCA3^+^ DC function, was excluded by Endolisa, a specific and sensitive method to detect endotoxins (data not shown). Next, we investigated whether HBsAg affected polyI:C-induced cytokine production. IFN-λ1-producing capacity, but not TNF-α-producing capacity, of both peripheral blood and intrahepatic BDCA3^+^ DCs upon polyI:C stimulation of PBMCs or liver cells, respectively, was significantly decreased by rHBsAg (Fig 3C–3E, data not shown). This effect was diminished upon depletion of rHBsAg by immunoprecipitation using anti-HBsAg-coated beads, and restored upon addition of rHBsAg to the depleted fraction (S5A Fig), indicating that the effect is HBsAg-specific. In addition, patient-derived HBsAg (pHBsAg) also reduced IFN-λ1 production by peripheral blood BDCA3^+^ DCs (S5B Fig). The HBsAg-induced maturation and functional impairment of BDCA3^+^ DCs, however, was only observed when these cells were exposed to HBsAg in the presence of other PBMCs. Incubation of isolated BDCA3^+^ DCs alone with rHBsAg neither induced DC maturation, nor affected polyI:C or TNFα and IL-1β-induced maturation, suggesting that HBsAg acts on BDCA3^+^ DCs only via other immune cells (Fig 4A). In addition, rHBsAg did not affect polyI:C-induced production of IL-1β, IL-6, IL-8, TNFα and IFN-λ by isolated BDCA3^+^ DCs and also had no effect on the viability of BDCA3^+^ DCs (Fig 4B and 4C, data not shown). Addition of PBMC to isolated BDCA3^+^ DCs increased the production of IFN-λ, which was reduced by rHBsAg, confirming that rHBsAg affected BDCA3^+^ DCs only indirectly (Fig 4C).

Together, these results demonstrate that HBsAg does not directly mature or impair BDCA3^+^ DC function, but may have an indirect effect via other immune cells.

## Discussion

BDCA3^+^ DCs are professional APCs that excel in IFN-λ production. In this study, we report on the intrahepatic presence and localization of BDCA3^+^ DCs in healthy and HBV-infected livers. We showed that BDCA3^+^ DCs reside in inflamed portal tracts and that their numbers are increased in HBV-infected livers compared to controls. In addition, blood BDCA3^+^ DCs of CHB patients displayed an impaired maturation and IFN-λ1 response upon ex vivo stimulation compared to controls. Furthermore, we demonstrated that the most prominent HBV protein, HBsAg, does neither directly induce BDCA3^+^ DC maturation, nor affects their function, but may exert an effect indirectly via an unknown mechanism.

Previous studies have shown that absolute numbers of BDCA1^+^ DCs and pDCs are increased in HBV-infected livers [32]. A result we here confirm and complement by demonstrating that also BDCA3^+^ DC numbers are increased in the liver upon HBV infection. IHC stainings showed that intrahepatic BDCA3^+^ DCs predominantly reside in portal tracts, and especially in those with high immune infiltration. Since these areas accommodate many other immune cells, including T cells, this suggests that BDCA3^+^ DCs may regulate immunity not only in the liver draining lymph nodes, but can also do so locally.

The impaired functional capacity of BDCA3^+^ DCs from CHB patients adds up to our previous findings for pDCs and mDCs, which demonstrated that the function of these DC subsets is also diminished in CHB patients [10, 11]. The reduced maturation capacity of BDCA3^+^ DCs together with a reduced IFN-λ production may impair T cell activation or skewing in these patients, and may thus affect the induction of effective adaptive immune responses [23].

We here find HBsAg was able to reduce IFN-λ production in vitro via an indirect mechanism. Therefore HBsAg may have a systemic effect that can contribute to the impaired IFN-λ production we observed in BDCA3^+^ DCs ex vivo, possibly via a monocyte-mediated mechanism [11, 33].

In contrast to the reduced maturation we observed for CHB patient-derived BDCA3^+^ DCs in response to polyI:C, maturation of BDCA3^+^ DCs in vitro was rather enhanced by HBsAg, suggesting the presence of alternative mechanisms and/or viral components that act on BDCa3+ DCs in these patients. Although BDCA3^+^ DCs may have become refractory to maturation as a result of continued HBsAg exposure, it is likely that the state of chronic inflammation itself plays a major role in reduction of BDCA3^+^ DC function as well. The exact contribution of viral proteins/particles or chronic inflammation in the impairment of BDCA3^+^ DC function during CHB remains to be determined.

Like blood BDCA3^+^ DCs, we and others showed that intrahepatic BDCA3^+^ DCs are able to produce IFN-λ [21]. In case of HCV infection, high IFN-λ levels have been detected in the liver, which may in part derive from BDCA3^+^ DCs [34]. CHB livers in contrast hardly contained IFN-λ transcripts [34]. Furthermore, IFN-λ levels in serum of CHB patients are comparable to those of controls [34–37]. One explanation may be that HBV by itself does not induce an effective IFN-λ response during its natural course of infection. However, our finding that IFN-λ1-producing capacity of BDCA3^+^ DCs is impaired in HBV patients, together with recent data showing the inhibition of IFN-λ production in infected hepatocytes by HBV virions, also open up the possibility that HBV may actively diminish IFN-λ1-production [38, 39]. These latter studies demonstrated that in hepatocytes, HBV can induce but concurrently suppress host innate responses, in particular the TLR3/RIG-I/MDA5-induced response, and that it does so by factors present in the viral inoculum and via pgRNA [38]. We here already demonstrate that HBsAg has an indirect effect on IFN-λ production by BDCA3^+^ DCs, but more research is required to find out how it does so and whether other viral factors and/or the state of chronic inflammation may also contribute to the defect observed in patients’ blood BDCA3^+^ DCs. A pressing remaining question now is whether in HBV-infected livers also intrahepatic BDCA3^+^ DCs show a reduced IFN-λ-producing capacity. Unfortunately, the scarceness of BDCA3^+^ DCs in the liver makes the functional experiments on biopsy BDCA3^+^ DCs extremely challenging. In addition, the availability of liver biopsies for such studies is limited, especially since implementation of the fibroscan to determine fibrosis stage. Therefore, performing such studies is at this moment beyond our possibility.

In conclusion, we demonstrate for the first time that BDCA3^+^ DCs are increased in HBV-infected livers and that the function of BDCA3^+^ DCs of HBV patients is impaired. These results suggest that BDCA3^+^ DCs are available on site to be exploited to improve/redirect HBV-specific immune responses. For example, by targeting local BDCA3^+^ DCs with TLR3 ligands to achieve local IFN-λ production, possibly even in combination with HBV antigens for simultaneous cross-presentation of viral antigens. To achieve optimal effect however, our study suggests that measures may need to be taken to overcome the impaired maturation and IFN-λ-producing capacity of BDCA3^+^ DCs in CHB patients.

## Supporting Information

S1 Fig Quantification of intrahepatic BDCA3^+^ DCs from HBV patients and controls. Liver cells were isolated from HBV patients and controls. The percentage BDCA3^+^ DCs of CD45^+^ cells (control n = 20, HBV n = 14) in livers of controls and HBV patients were determined. Indicated are the mean percentage and SEM. Open dots represent cells from donor livers and filled dots represent cells from peri-tumor liver tissue.
(TIF)

S2 Fig Specific expression of CLEC9A on BDCA3^+^ DC. Representative flow cytometry plots of CLEC9A expression on lineage^-^CD45^+^HLA-DR^+^BDCA3^hi^ liver cells.
(TIF)

S3 Fig Upregulation of maturation markers upon stimulation. PBMC were isolated from CHB patients and healthy controls and stimulated for 5 hours with or without polyI:C. Expression of the maturation markers CD40, CD83 and CD86 by BDCA3^+^ DCs was measured by flow cytometry. Collected expression data (MFI) for each marker after isolation at t = 0 and stimulation at t = 5 is shown (control n = 15, HBV n = 8) (mean±SEM).
(TIF)

S4 Fig Correlation between IFN-λ and TNF-α production. PBMCs of CHB patients and healthy controls were stimulated for 5 hours with or without polyI:C. (A) Representative histogram including the isotype control of IFN-λ1 production by FSC/SSC-gated viable BDCA3^+^ DCs. (B) Representative flow cytometry plots of TNF-α and IFN-λ1 production by FSC/SSC-gated viable BDCA3^+^ DC. (C) Pearson’s correlation between TNF-α and IFN-λ1 production by BDCA3^+^ DCs from controls (open dots) and HBV patients (filled dots) (n = 28).
(TIF)

S5 Fig Inhibition of IFN-λ production by pHBsAg and rHBsAg. (A) PBMCs of healthy controls were stimulated for 7 hours with polyI:C in the presence or absence of rHBsAg (HBsAg), a fraction from which rHBsAg was depleted (α-HBs-Ig treated), or a HBsAg-depleted fraction to which (5 μg ml^-1^) rHBsAg was added (α-HBs-Ig treated + HBsAg). The mean ±SEM percentage of IFN-λ1-producing BDCA3^+^ DCs is shown. * p<0.05 by paired Student’s *t*-test (B) PBMC of healthy controls were stimulated for 7 hours with or without polyI:C in the presence or absence of pHBsAg. The production of IFN-λ1 by BDCA3^+^ DCs was measured by ICS. The mean±SEM percentage of IFN-λ1 BDCA3^+^ DCs from 5 different donors is shown. * p<0.05 by paired Student’s *t*-test.
(TIF)
