# Supplementary figures and images for: The Effect of Chronic Hepatitis B Virus Infection on BDCA3+ Dendritic Cell Frequency and Function
Source: PLoS One. 2016 Aug 16;11(8):e0161235. doi: 10.1371/journal.pone.0161235 (PMC4987041; doi:10.1371/journal.pone.0161235)

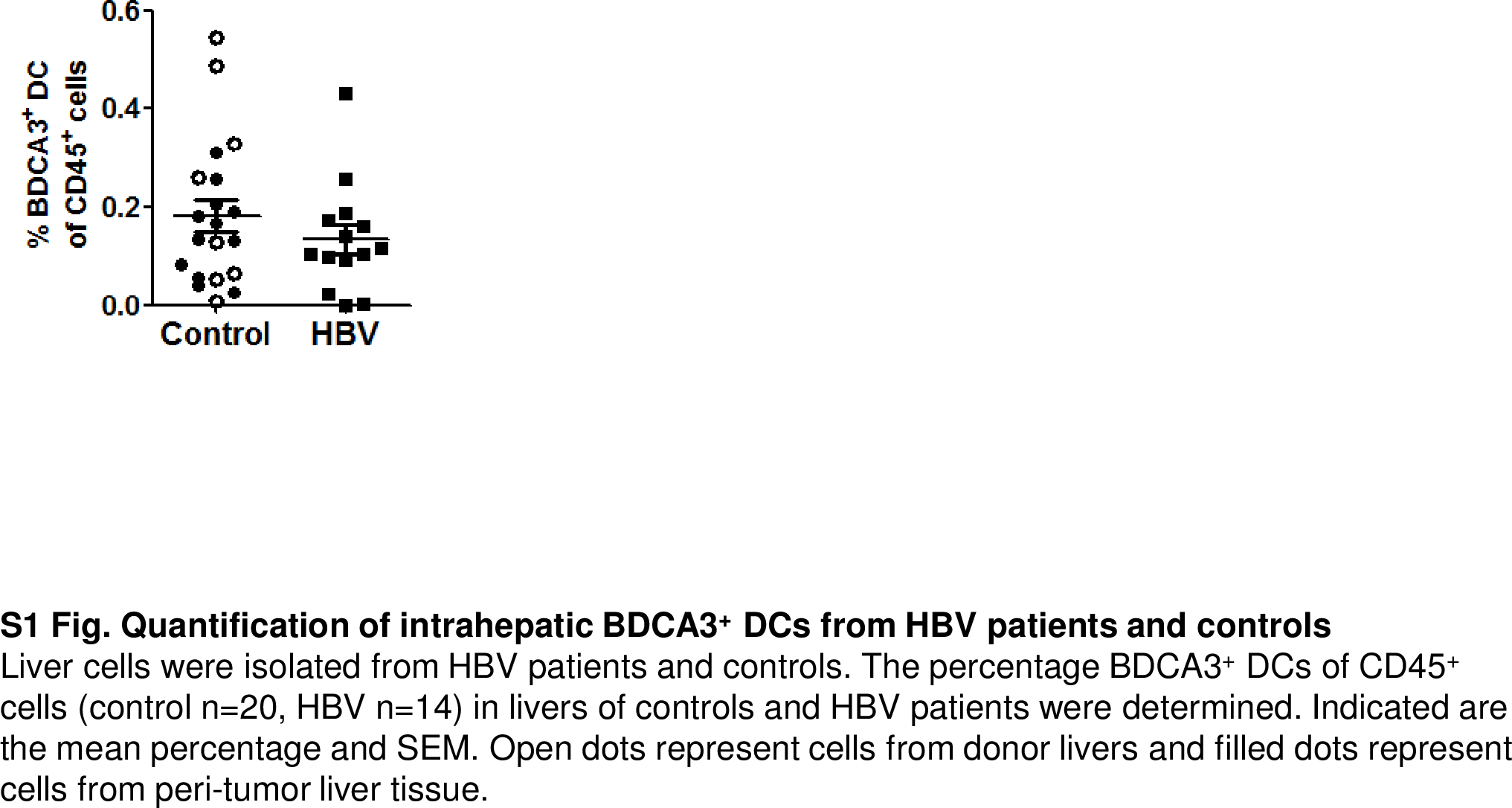

Supplement: S1 Fig — Liver cells were isolated from HBV patients and controls. The percentage BDCA3+ DCs of CD45+ cells (control n = 20, HBV n = 14) in livers of controls and HBV patients were determined. Indicated are the mean percentage and SEM. Open dots represent cells from donor livers and filled dots represent cells from peri-tumor liver tissue. (TIF) [file pone.0161235.s001.tif]

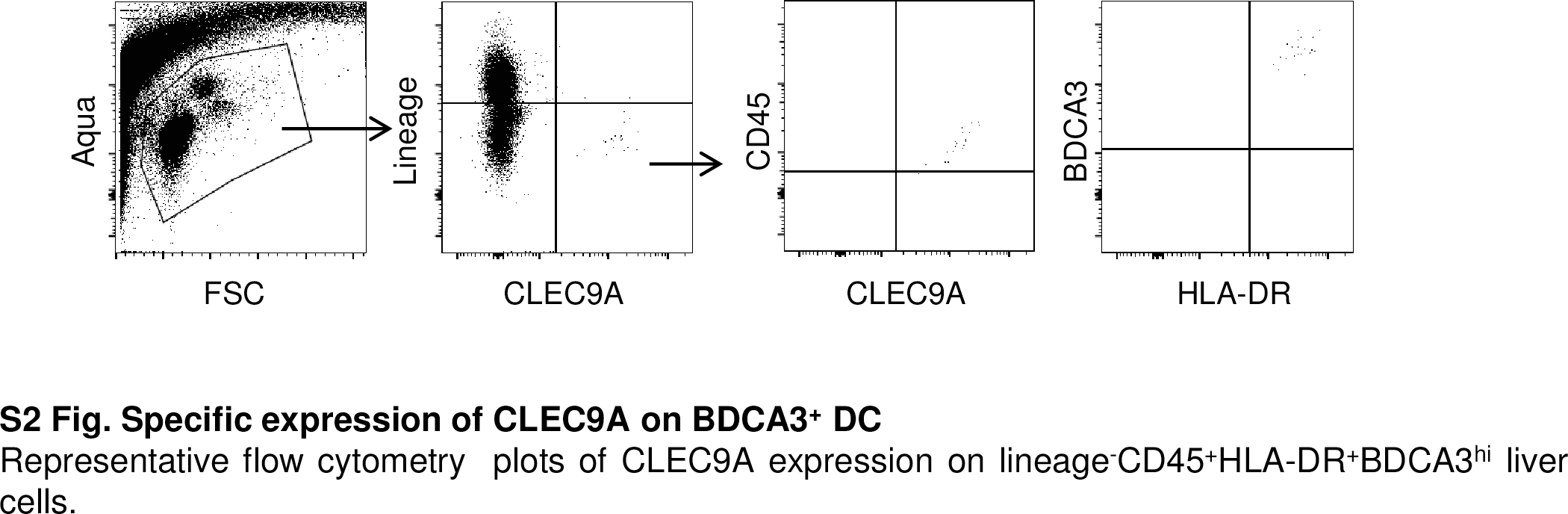

Supplement: S2 Fig — Representative flow cytometry plots of CLEC9A expression on lineage-CD45+HLA-DR+BDCA3hi liver cells. (TIF) [file pone.0161235.s002.tif]

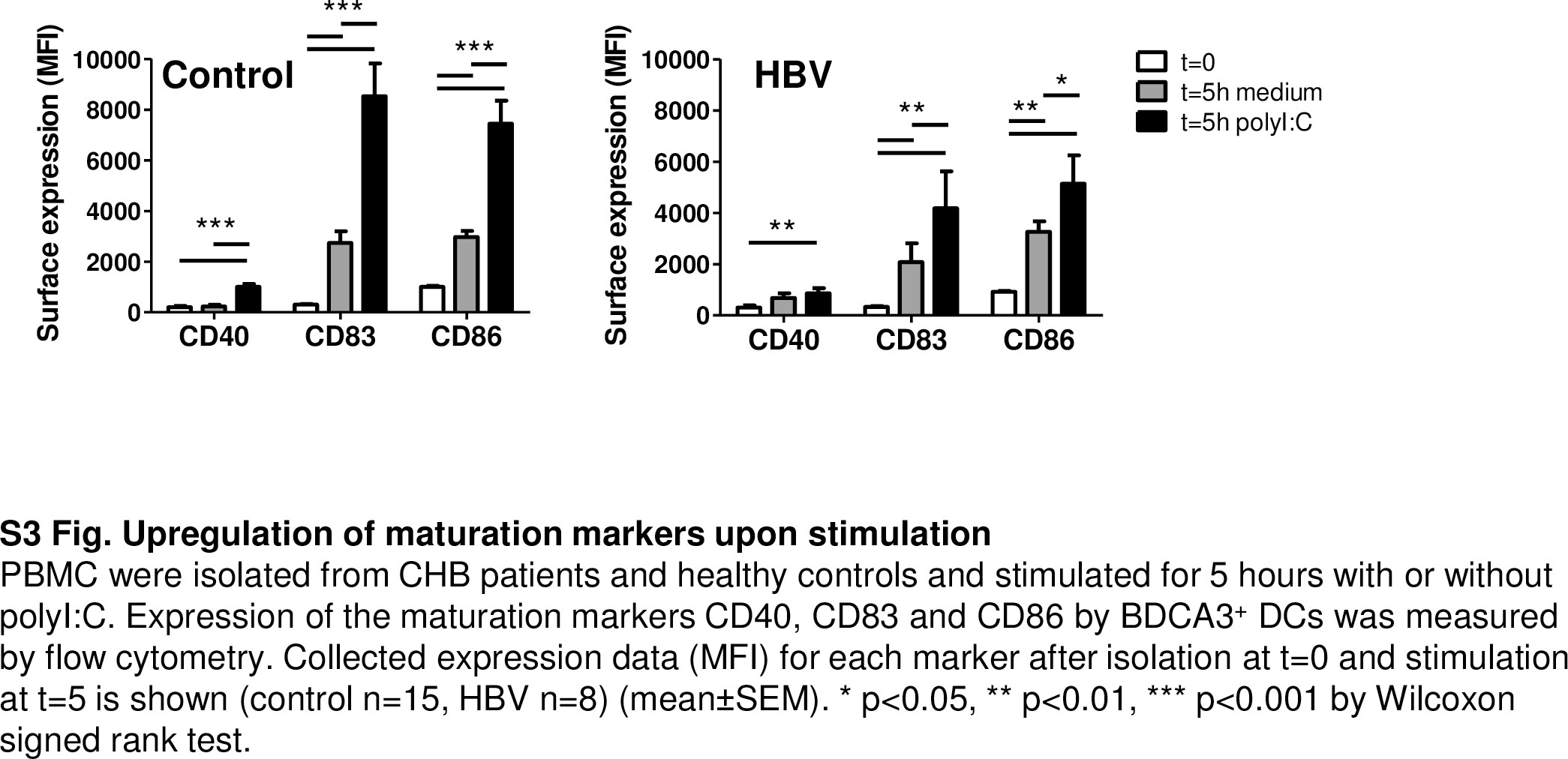

Supplement: S3 Fig — PBMC were isolated from CHB patients and healthy controls and stimulated for 5 hours with or without polyI:C. Expression of the maturation markers CD40, CD83 and CD86 by BDCA3+ DCs was measured by flow cytometry. Collected expression data (MFI) for each marker after isolation at t = 0 and stimulation at t = 5 is shown (control n = 15, HBV n = 8) (mean±SEM). (TIF) [file pone.0161235.s003.tif]

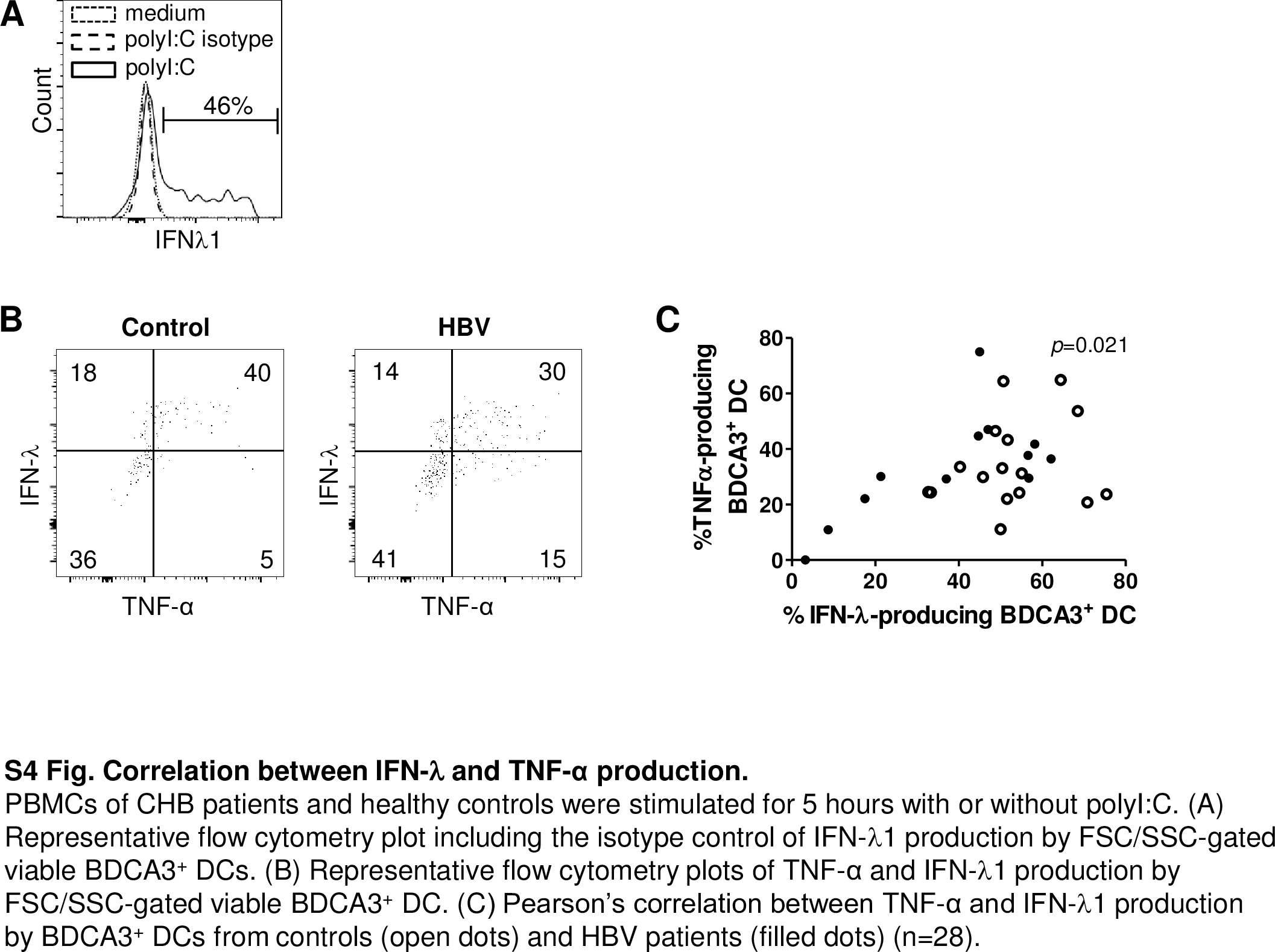

Supplement: S4 Fig — PBMCs of CHB patients and healthy controls were stimulated for 5 hours with or without polyI:C. (A) Representative histogram including the isotype control of IFN-λ1 production by FSC/SSC-gated viable BDCA3+ DCs. (B) Representative flow cytometry plots of TNF-α and IFN-λ1 production by FSC/SSC-gated viable BDCA3+ DC. (C) Pearson’s correlation between TNF-α and IFN-λ1 production by BDCA3+ DCs from controls (open dots) and HBV patients (filled dots) (n = 28). (TIF) [file pone.0161235.s004.tif]

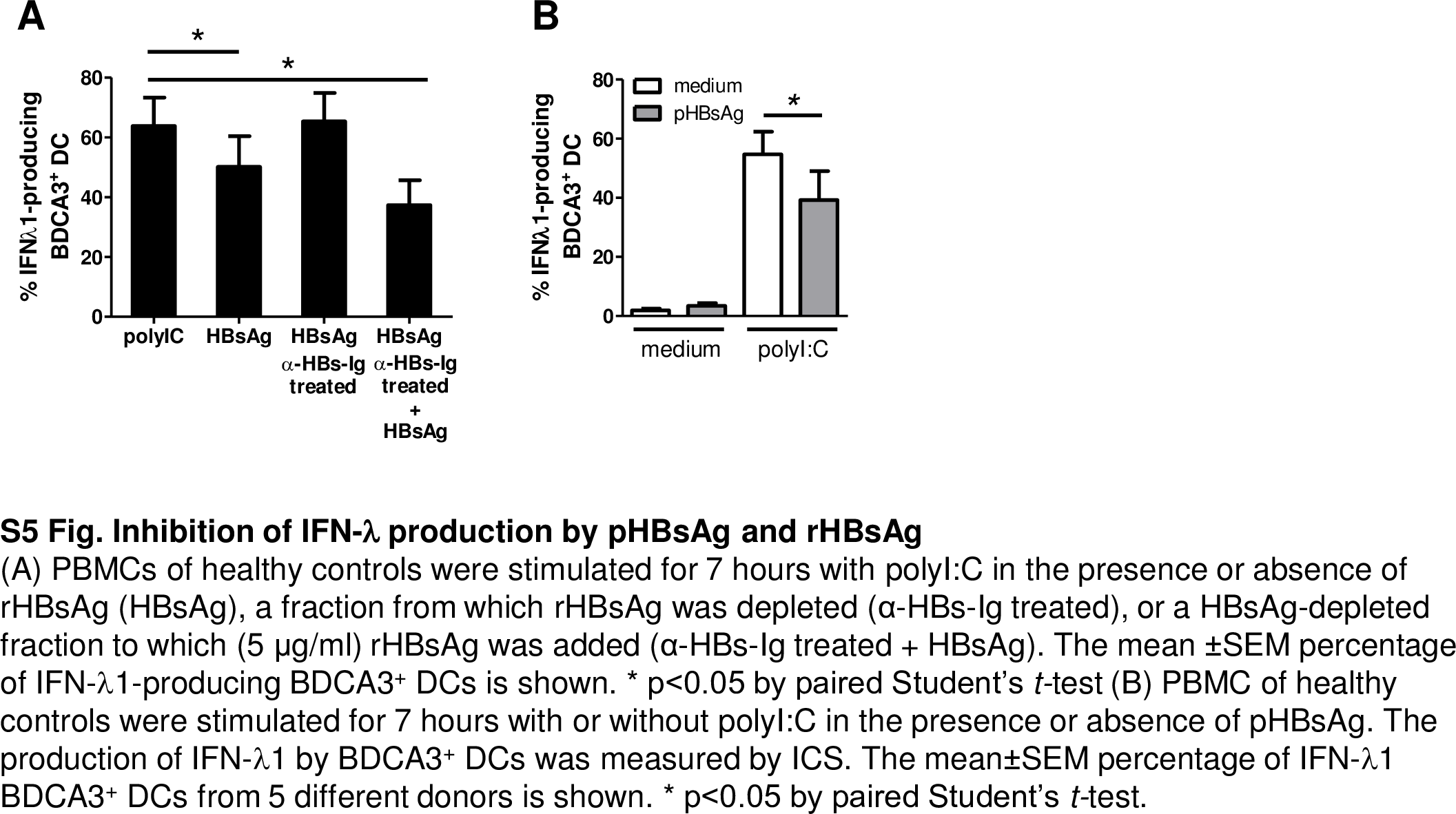

Supplement: S5 Fig — (A) PBMCs of healthy controls were stimulated for 7 hours with polyI:C in the presence or absence of rHBsAg (HBsAg), a fraction from which rHBsAg was depleted (α-HBs-Ig treated), or a HBsAg-depleted fraction to which (5 μg ml-1) rHBsAg was added (α-HBs-Ig treated + HBsAg). The mean ±SEM percentage of IFN-λ1-producing BDCA3+ DCs is shown. * p<0.05 by paired Student’s t-test (B) PBMC of healthy controls were stimulated for 7 hours with or without polyI:C in the presence or absence of pHBsAg. The production of IFN-λ1 by BDCA3+ DCs was measured by ICS. The mean±SEM percentage of IFN-λ1 BDCA3+ DCs from 5 different donors is shown. * p<0.05 by paired Student’s t-test. (TIF) [file pone.0161235.s005.tif]
